# Tospoviruses Induce Small Interfering RNAs Targeting Viral Sequences and Endogenous Transcripts in Solanaceous Plants

**DOI:** 10.3390/pathogens11070745

**Published:** 2022-06-30

**Authors:** Stephen J. Fletcher, Jonathan R. Peters, Cristian Olaya, Denis M. Persley, Ralf G. Dietzgen, Bernard J. Carroll, Hanu Pappu, Neena Mitter

**Affiliations:** 1 Centre for Horticultural Science, Queensland Alliance for Agriculture and Food Innovation, The University of Queensland, St. Lucia, Brisbane, QLD 4072, Australia; s.fletcher@uq.edu.au (S.J.F.); jonathan.peters@uq.edu.au (J.R.P.); r.dietzgen@uq.edu.au (R.G.D.); 2 Department of Plant Pathology, Washington State University, Pullman, WA 99164-6430, USA; cristian.olayaarias@wsu.edu; 3 Queensland Department of Agriculture and Fisheries, AgriScience Queensland, EcoSciences Precinct, Dutton Park, Brisbane, QLD 4102, Australia; denis.persley@daf.qld.gov.au; 4 School of Chemistry and Molecular Biosciences, The University of Queensland, St. Lucia, Brisbane, QLD 4072, Australia; b.carroll@uq.edu.au

**Keywords:** capsicum chlorosis virus, tomato spotted wilt virus, tospovirus, solanaceae, virus activated short-interfering RNA, viral short-interfering RNA, RNA interference, vasiRNA

## Abstract

Tospoviruses infect numerous crop species worldwide, causing significant losses throughout the supply chain. As a defence mechanism, plants use RNA interference (RNAi) to generate virus-derived small-interfering RNAs (vsiRNAs), which target viral transcripts for degradation. Small RNA sequencing and in silico analysis of capsicum and *N. benthamiana* infected by tomato spotted wilt virus (TSWV) or capsicum chlorosis virus (CaCV) demonstrated the presence of abundant vsiRNAs, with host-specific differences evident for each pathosystem. Despite the biogenesis of vsiRNAs in capsicum and *N. benthamiana*, TSWV and CaCV viral loads were readily detectable. In response to tospovirus infection, the solanaceous host species also generated highly abundant virus-activated small interfering RNAs (vasiRNAs) against many endogenous transcripts, except for an *N. benthamiana* accession lacking a functional *RDR1* gene. Strong enrichment for ribosomal protein-encoding genes and for many genes involved in protein processing in the endoplasmic reticulum suggested co-localisation of viral and endogenous transcripts as a basis for initiating vasiRNA biogenesis. RNA-seq and RT-qPCR-based analyses of target transcript expression revealed an inconsistent role for vasiRNAs in modulating gene expression in *N. benthamiana*, which may be characteristic of this tospovirus-host pathosystem.

## 1. Introduction

Tospoviruses are taxonomically classified in the genus *Orthotospovirus*, family *Tospoviridae*, order *Bunyavirales* [1]. In targeting more than 1090 species in 85 families of monocotyledonous and dicotyledonous plants, tospoviruses cause severe production losses in crops worldwide [2]. Capsicum chlorosis virus (CaCV) and tomato spotted wilt virus (TSWV) are two tospoviruses of significance across Asia and Australia, impacting many horticultural species in warmer climates [3]. Thrips (*Frankliniella* and *Thrips* species) are the primary transmission vectors for these tospoviruses and are also replication hosts, though without associated symptoms [4].

Both CaCV and TSWV have single-stranded RNA genomes that comprise five open reading frames across three segments, with one negative-sense and two ambisense. The small (S) RNA encodes a non-structural RNA-silencing suppressor protein (NSs) and the nucleocapsid protein (N). The medium (M) RNA encodes a cell-to-cell movement protein (NSm) and the precursor of the two envelope glycoproteins (GP), with the Large (L) RNA coding for an RNA-directed RNA polymerase (RdRp), also called L protein [5,6]. 

Viral RNAs act as both a substrate for and target of the host plant’s RNA interference (RNAi) defence pathways, leading to viral lifecycle disruption through specific transcript degradation. Double-stranded RNAs (dsRNAs) arising from processes such as viral replication are cleaved by Dicer-like enzymes into virus-derived small-interfering RNA (vsiRNA) duplexes of primarily 21 and 22 nucleotides (nt) [7]. VsiRNAs are then bound by AGO proteins as part of the RNA-induced silencing complex (RISC), which subsequently degrades complementary transcripts. Once initiated, secondary vsiRNAs can also be generated via the actions of RNA-dependent RNA polymerases (RDRs), with an associated amplification of silencing through second-strand synthesis from a single-stranded RNA template. Generation of vsiRNAs in response to TSWV infection has previously been demonstrated in multiple plant hosts, including peanut (*Arachis hypogaea*; Fabaceae), tomato (*Solanum lycopersicum*; Solanaceae), and *Nicotiana benthamiana* (Solanaceae) [8,9,10]. 

As well as targeting viral transcripts, the plant’s RNAi machinery has several roles in endogenous gene regulation, acting both transcriptionally and post-transcriptionally. Recently, multiple studies have revealed that virus infection triggers the biogenesis of siRNAs targeting host transcripts (termed virus-activated small interfering RNAs, vasiRNAs), in addition to vsiRNAs in *Arabidopsis thaliana* [11,12,13]. VasiRNA biogenesis was observed following inoculation with a cucumber mosaic virus (CMV) mutant carrying a non-functional 2b suppressor of RNA silencing gene and additionally with turnip mosaic virus (TuMV), which encodes a weak silencing suppressor. The 2b protein is thought to reduce vsiRNA abundance by direct sequestration of these siRNA species [14]. Though RDR6, along with RDR1, amplify the RNAi response through the generation of dsRNA from a viral RNA template, vasiRNA production appears to be exclusively RDR1-dependent. Gou and collaborators demonstrated that the P-type ATPases ALA1 and ALA2, which transport phospholipids across cellular membranes, are also essential for vasiRNA production [12]. The authors suggested ALA1 and ALA2 have a role in the formation of vesicle-like membrane invaginations around dsRNA precursors. Further work by the same group identified Antiviral RNAi-defective 2 (AVA2), a putative magnesium transporter, as an additional requirement for vasiRNA biogenesis. AVA2 is also localised to the endoplasmic reticulum (ER), similar to ALA1 and ALA2 [13]. More recently, vasiRNAs have been detected in TuMV infection sites in *Brassica napus* leaves, with demonstrated roles in cleavage of host as well as viral transcripts [15]. Interestingly, infection of Arabidopsis, *Brassica rapa*, and *Brassica napus* by cauliflower mosaic virus (CaMV), a double-stranded DNA virus, also produced vasiRNAs [16]. Target host transcripts identified in that study functionally overlapped with those previously identified by Cao and collaborators for CMV infection, with roles in photosynthesis and stress response [11,16]. 

Following their identification and characterisation in the Brassicaceae, the search for vasiRNAs was extended to solanaceous species. Initially, a lack of detectable vasiRNAs in TSWV-infected *N. benthamiana* was reported [17]. The authors noted that the presence or absence of a functional NSs protein did not impact vasiRNA abundance and speculated that either a defective RDR1 in the *N. benthamiana* accession used or differences between CMV 2b and TSWV NSs vasiRNA sequestration might be responsible. Using a transgenic approach, the addition of a functional RDR1 to the *N. benthamiana* accession used resulted in the generation of vasiRNAs following tomato leaf curl Gujarat virus infection, and as with the Brassicaceae species, differential regulation of target genes [18]. 

In this study, vsiRNA and vasiRNA responses to TSWV and CaCV infection were examined using next-generation sequencing of solanaceous species including *N. benthamiana* and capsicum (*Capsicum annum*). *N. benthamiana* varieties with and without the ability to generate vasiRNAs were used to shed light on the role of vasiRNAs in disease progression as well as endogenous host gene regulation. For each of the species examined, abundant vsiRNA biogenesis against both TSWV and CaCV was evident. In hosts with a functional RDR1, vasiRNA generation was also robust, with localisation of substrate host transcripts proposed as a selective mechanism. Interestingly, the generation of vasiRNAs in *N. benthamiana* did not result in a general downward trend in target host gene expression attenuation, suggesting many competing factors impact the expression of these genes during infection. 

## 2. Materials and Methods

### 2.1. Host Plants and Tospovirus Isolates 

CaCV isolate QLD-3432, provided by the Queensland Department of Agriculture and Fisheries (DAF), was maintained in capsicum (*Capsicum annuum*) cv. Yolo Wonder, with the genome of this isolate previously sequenced [19]. TSWV isolate QLD-1 infected capsicum samples were also provided by DAF. This isolate was collected from Bowen, the same region as an Australian isolate that was recently sequenced [20]. *C. annuum* cv. Yolo Wonder, *Nicotiana benthamiana* LAB, and *N. benthamiana* Western Australia (WA; provided by Peter Waterhouse laboratory at the Queensland University of Technology) were used as host plants. All plant material described here was used for TSWV-infection and/or CaCV-infection assays, small RNA sequencing, and RNA-seq.

In this study, small RNA read data from susceptible (Marglobe) and resistant (Red Defender) tomato varieties following TSWV infection were also reanalysed. Red Defender’s resistance is associated with hypersensitivity induced by the *Sw-5* gene [21]. The generation of this read data, including plant growth conditions, inoculation methodology, symptoms, and extraction of total RNA, has previously been described [9]. 

### 2.2. Virus Inoculations

Capsicum at the four-leaf stage and *N. benthamiana* at the five-six leaf stage were mechanically inoculated with homogenates from TSWV (QLD-1) or CaCV (QLD-3432) infected plant tissue of the same species. For mechanical inoculation, one or two apical leaves from a systemically infected symptomatic plant were homogenised with chilled mortar and pestle in 10 mM sodium phosphate buffer (pH 7.0) containing 0.4% 2-mercaptoethanol and rub-inoculated to carborundum-dusted leaves of host/test plants.

The youngest two leaves of capsicum plants and leaves two and three of *N. benthamiana* plants were virus-inoculated. As a negative control, sap from non-infected plants was used for mechanical inoculation (mock treatment). At least three plants were used for each treatment. Virus infection of plants was confirmed by the progression of symptoms, qPCR or DAS-ELISA using TSWV or CaCV kits from Agdia, Inc. (Elkhart, IN, USA) and following the manufacturer’s instructions. Plant images showing symptoms were processed using Adobe Lightroom Classic (v11.4, Adobe, San Jose, CA, USA) to ensure the evenness of exposure and white balance. 

### 2.3. RNA Extraction and Quality Assessment

For all capsicum and *N. benthamiana* treatments, apical leaf tissue was collected ten days after mechanical inoculation, snap-frozen in liquid nitrogen, and stored at −80 °C. Tissue was subsequently ground with a chilled mortar and pestle, and total RNA was extracted using TRIsure™ reagent (Bioline, Memphis, TN, USA), following the manufacturer’s instructions. RNA samples were suspended in nuclease-free water, and the quantity and purity were determined using a NanoDrop™ spectrophotometer (ThermoFisher, Waltham, MA, USA). The quality of the RNA was assessed by the Australian Genome Research Facility (AGRF, Brisbane, Australia) using a Bioanalyzer (Agilent, Santa Clara, CA, USA). 

### 2.4. Sequencing Methods

Capsicum and *N. benthamiana* RNA samples were submitted to AGRF for small RNA sequencing. Small RNA libraries were prepared using the New England Biolabs (Ipswich, UK) kit protocol and selecting for RNA of 18–33 nucleotides (nt). Illumina HiSeq HT Chemistry was used with 50 bp single reads. Samples were distributed across three flow cells. Small RNA sequencing of tomato samples has previously been described [9].

To examine differential expression of vasiRNA targets, RNA-seq was performed on total RNA from apex tissues of *N. benthamiana* LAB and WA accessions 10 days after TSWV or mock inoculation. RNA-seq of *N. benthamiana* samples was conducted by Genewiz, with non-strand specific library preparation carried out prior to generation of approximately 20 M paired-end reads per sample on a NovaSeq platform. Raw sequence data is available at the NCBI Sequence Read Archive under BioProject ID PRJNA784690.

### 2.5. Bioinformatic Analysis

Small RNA deep sequencing read files were processed with the FASTX-Toolkit (http://hannonlab.cshl.edu/fastx_toolkit/, accessed on 1 December 2021) to remove 5′ adapters and collapse reads to unique sequence:count couplets. Reads were aligned to TSWV and CaCV genome segments and endogenous transcript sequences using the SCRAM aligner [22]. Profile and comparison plots were generated using the SCRAM pipeline, with differential vasiRNA read abundance calculated using DeSeq2. Boxplots and Venn diagrams were generated using R and Python, respectively. Tomato small RNA sequence data was obtained from the NCBI SRA (BioProject PRJNA606610) and processed using the same bioinformatics pipeline. Large RNA-seq reads were trimmed with Trim Galore (https://github.com/FelixKrueger/TrimGalore, accessed on 1 December 2021), and then vasiRNA-target transcript abundance was quantified with Kallisto [23]. Plots were generated using Python scripts. Genome alignments of short reads were carried out using Bowtie [24], with no mismatches allowed.

KEGG pathway analysis involved identifying transcripts that had significant differences in complementary vasiRNAs (*p* < 10^−8^) for TSWV-infected and mock-inoculated capsicum, tomato (Marglobe), and *N. benthamiana* (WA genotype) samples. For each differentially expressed (DE) transcript, the longest open reading frame (ORF) was identified and translated to a peptide using a custom Python script. Peptides were assigned KEGG identifiers using BLASTKoala [25]. These identifiers, along with log-fold change values for each species, were analysed using Pathview [26], which classified the ‘protein processing in ER’ and ‘ribosome’ pathways as significantly enriched for vasiRNA DE transcripts. Figures were generated by Pathview with custom colour and limit settings.

### 2.6. RT-qPCR Analysis

RNA samples were first treated with TURBO DNase I™ (ThermoFisher, Waltham, MA, USA) and reactions terminated with 15 mM EDTA. cDNA was synthesised using the sensiFAST™ cDNA synthesis kit with 1.88 mM MgCl_2_ added to sequester additional EDTA from DNase treatment (Bioline, Memphis, TN, USA). Changes in transcript abundance were calculated for mock vs. infected samples as −ΔΔCt to meet the assumptions of the statistical tests used. As an RT- control to rule out genomic DNA contamination, RNA samples underwent PCR with MyTaq™ DNA polymerase (Bioline, Memphis, TN, USA), with no amplification evident. Primers used for qPCR are shown in Table S2.

## 3. Results

### 3.1. Tospovirus Infection of Solanaceous Plants

Responses to CaCV or TSWV infection were investigated in two host plant species of the Solanaceae family: capsicum and *N. benthamiana*. Of the two *N. benthamiana* genotypes, *N. benthamiana* WA had previously unknown TSWV susceptibility, and *N.* *benthamiana* LAB with a non-functional RDR1 is considered susceptible to many plant viruses, including TSWV [27,28]. 

Following TSWV or CaCV inoculation in capsicum, mild leaf mottling and yellowing symptoms were evident compared to mock-inoculated plants at 10 dpi (Figure S1C). CaCV and TSWV RNA accumulation in non-inoculated apical leaf tissue was measured by RT-qPCR and confirmed systemic virus infection of inoculated plants (Figure S5A). 

Like tospovirus-challenged capsicum plants, *N. benthamiana* LAB showed mild symptoms with CaCV or TSWV infection compared to the mock treatment (Figure S1A). Correspondingly, the symptoms of *N. benthamiana* WA infected with TSWV were also mild compared to mock treatments (Figure S1B). Both the *N. benthamiana* WA and LAB accessions showed mild leaf curling and mottling (Figure S1A,B). *N. benthamiana* WA and LAB plants also had similar viral titres as assessed by RT-qPCR of the *N* gene and ELISA (Figure S5B,C). However, at 23 dpi, *N. benthamiana* LAB plants appeared yellow and perished with signs of necrosis at the apex, whereas *N. benthamiana* WA and capsicum plants survived with new green leaf tissue emerging (Figure S2). At 47 dpi, LAB plants had completely perished, while WA plants generally survived. 

### 3.2. Tospovirus Infection Induces a Shift in Small RNA Size Distribution 

**Figure 1:**
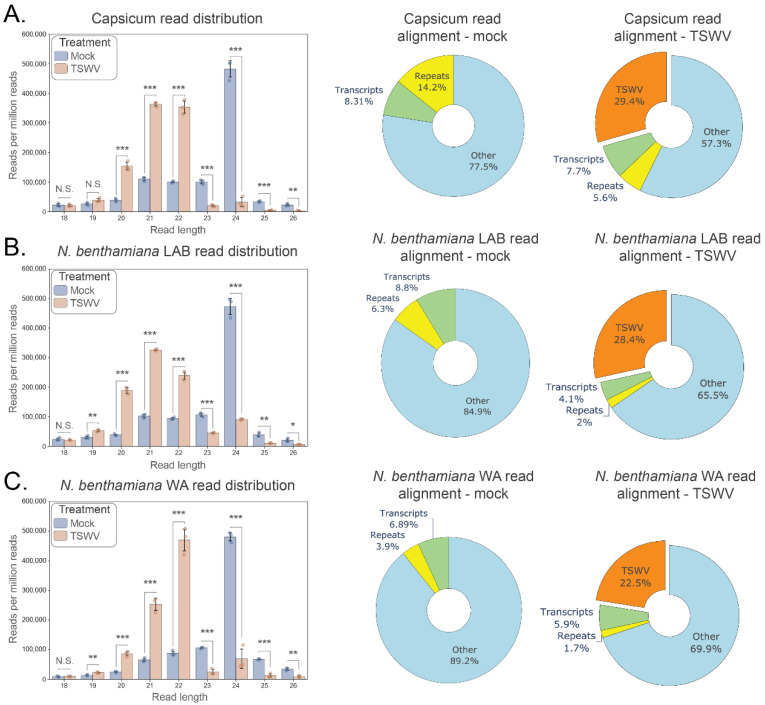
Changes in small RNA read distribution in solanaceous hosts following inoculation by TSWV. Small RNA read distributions are shown for each host 10 days post tomato spotted wilt virus (TSWV) and mock inoculation (*n* = 3 for each treatment). (**A**). Capsicum, (**B**). *Nicotiana benthamiana* LAB, (**C**). *N*. *benthamiana* WA. Left panels: Small RNA read distribution by length (18–26 nt; error bars represent ± 1 SD; individual data points shown); Right panels: host and TSWV functional genomic alignment of reads for mock and TSWV treatments (pie chart percentages are the average of three replicates aligning to host transcripts, host repeat elements, other host genomic locations, and the combined RNA segments of TSWV). For each host, a significant reduction in the relative abundance of 23–24 nt reads and increase in 20–22 nt reads has occurred 10 days post TSWV inoculation (*t*-test; 2 tailed; FDR-BH multiple correction; N.S. (not significant): *p* ≥ 0.05, *: *p* < 0.05, **: *p* < 0.01, ***: *p* < 0.001). The proportion of reads aligning to TSWV genome segments ranges from an average of 22.5–29.4% (orange; right pie chart) at this timepoint, indicating a sizable small RNA perturbation in response to TSWV infection.

Small RNA sequencing was conducted on TSWV or CaCV infected capsicum and *N. benthamiana* samples to help elucidate the molecular response of these solanaceous species to tospovirus infection. In silico analyses of the resulting sequence data revealed dramatic changes in the relative abundance of 20 to 24 nt reads in both *N. benthamiana* accessions as well as capsicum in response to TSWV (Figure 1) or CaCV (Figure S3A,B) infection. In mock treatments, 24 nt reads were most abundant, whereas 21 to 22 nt reads were dominant in tospovirus-infected samples. An average of 29.4%, 28.4%, and 22.5% of the total small RNA reads from TSWV-inoculated capsicum, *N. benthamiana* LAB, and WA, respectively, perfectly matched the TSWV genome, and 43.7% and 12.1% of total small RNAs from CaCV-infected capsicum and *N. benthamiana* LAB, respectively, targeted the CaCV genome (*n* = 3 biologically independent replicates per treatment; Figure S3A,B). Accompanying the shift towards 21–22 nt reads was a reduction in reads mapping to host repeated elements. 

Dicer-like (DCL) enzymes process double-stranded RNA into 21 and 22 nt duplexes [29]. Due to the observed shift in small RNA size classes, expression levels of four *DCL* homologues in TSWV-and CaCV-infected capsicum and *N. benthamiana* were first analysed using RT-qPCR (Figure S5D–F). The general response of *DCL* upregulation was evident in response to TSWV or CaCV in both capsicum and *N. benthamiana*, though, in one repeated qPCR experiment, *DCL2* was downregulated in *N. benthamiana WA*, which may be a result of experiment-to-experiment variation. Expression of these genes was also examined in *N. benthamiana* using RNA-seq, which showed upregulation in response to TSWV infection for *DCL1*, *DCL2*, and *DCL4*, and downregulation of *DCL3* in *N. benthamiana* Lab and WA (Figure S6F–I). 

### 3.3. Hotspots of vsiRNA Abundance Are Evident for TSWV and CaCV

**Figure 2:**
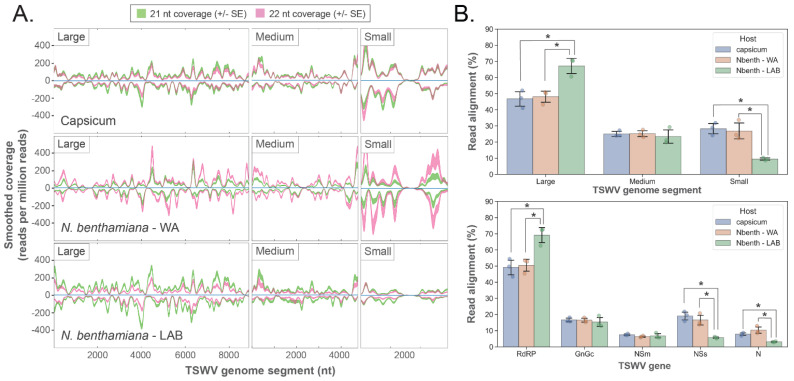
Mapping of 21 and 22 nt reads to TSWV RNA segments and genes reveals alignment hotspots and host-specific variation. Small RNA reads were generated from samples 10 days post TSWV inoculation (*n* = 3 for each host; mock data not shown as aligning reads absent). (**A**). Small RNA coverage profiles for the Large, Medium, and Small TSWV RNA segments. Each plot shows smoothed read coverage in both orientations (shaded regions represent ± 1 SE for each alignment length). Consistently located hotspots of increased coverage (prominent peaks) are evident in each segment and in each orientation for all hosts. (**B**). Mean alignment proportions to TSWV RNA segments and genes for each host (*n* = 3; error bars represent ± 1 SD; individual data points shown). Significantly more reads aligned to the large TSWV RNA segment in *N. benthamiana* LAB compared to WA and capsicum, and fewer reads aligned to the small segment for the same comparison (top). Small RNA alignments to TSWV genes showed a similar pattern, with more reads aligning to RdRP in *N. benthamiana* LAB compared to the other hosts and fewer reads to the *NSs* and *N* genes in the same comparison (bottom). RdRP covers most of the large RNA segment, and only NSs and N are located in the small RNA segment. (*t*-test; 2-tailed; FDR-BH multiple correction; *: *p*<0.05).

Infection of capsicum and *N. benthamiana* by TSWV (Figure 2) or CaCV (Figure S3C,D) resulted in the biogenesis of vsiRNAs. For TSWV infected samples, in silico small RNA sequence analysis showed ‘hotspots’ of vsiRNA abundance (easily discernible peaks in vsiRNA coverage) that spanned all three genome segments in both the sense and antisense orientations (Figure 2A). A slightly greater total abundance of 22 nt relative to 21 nt siRNAs was apparent for *N. benthamiana* WA, but in contrast, 21 nt vsiRNAs were more abundant than 22 nt vsiRNAs for capsicum and *N. benthamiana* LAB. The percentage of vsiRNA reads that aligned to each RNA segment and gene transcript showed significant differences between the two *N. benthamiana* accessions; the L segment comprising the RdRP gene had a significantly greater percentage of aligning vsiRNAs in the LAB than the WA accession, with a corresponding reduction of vsiRNAs aligning with the S segment and *NSs* and *N* genes (Figure 2B). There were no significant differences between alignments to genome segments or genes for the capsicum and *N. benthamiana* WA hosts. In response to CaCV infection, vsiRNA hotspots were also evident across the three segments, and there were significant differences between capsicum and *N. benthamiana* LAB for alignments to the M and S segments and the genes present in these segments (Figure S3C,D). 

### 3.4. Virus-Activated siRNAs Associated with Endogenous Transcripts in Solanaceous Plant Species

**Figure 3:**
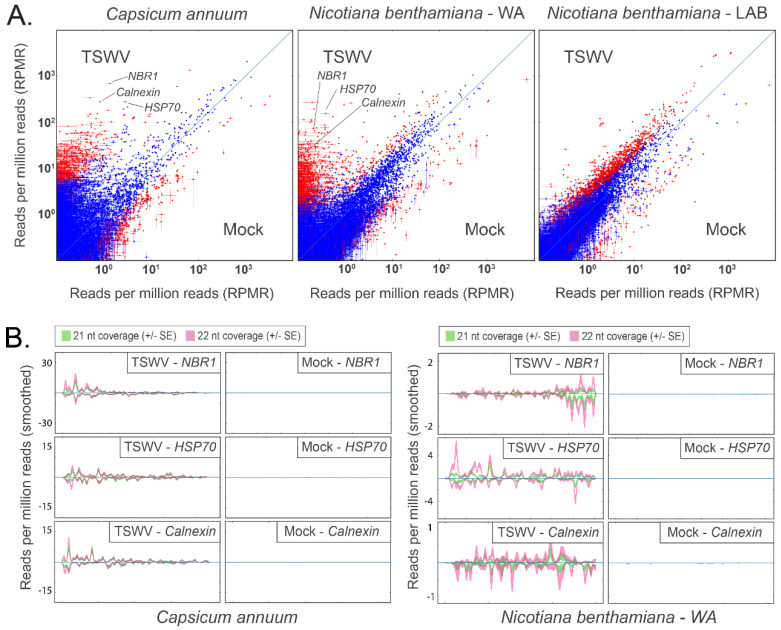
Host-specific alignment of 21 nt and 22 nt reads to host transcripts 10 days post TSWV inoculation suggests the presence of vasiRNAs in capsicum and *N. benthamiana* WA, but not in *N. benthamiana* Lab. These alignments are largely absent in mock-inoculated samples. (**A**) Comparison plots for transcriptome-wide alignments to capsicum (left), *N. benthamiana* WA (centre), and *N. benthamiana* Lab (right) are shown. Each point indicates the normalised alignment abundance in TSWV-inoculated samples (Y-axis) and mock inoculated samples (X-axis) to each transcript (*n* = 3 for each treatment; vertical and horizontal bars represent ± 1 SE). Red points show a significant difference between the alignment abundance of each treatment (TSWV v. mock), whereas blue points are not significant (calculated using DESeq2; red: *p*_adj_ < 10^−8^, blue: *p*_adj_ > 10^−8^). *NBR1*, *HSP70*, and *Calnexin* (individually labelled) are examples of transcripts with abundant 21 nt and 22 nt alignments in TSWV-infected hosts. (**B**) Profile plots for vasiRNA targets *NBR1*, *HSP70*, and *Calnexin*. Furthermore, 21 nt and 22 nt alignment hotspots are evident in TSWV-inoculated capsicum and *N. benthamiana* WA samples but absent in mock-inoculated samples. Hotspots occur in both orientations, indicating the originating template was dsRNA. Profile alignments were absent in *N. benthamiana* Lab (data not shown).

In silico analysis of small RNA reads exactly matching host transcripts suggested vasiRNAs were generated in response to TSWV and CaCV infection in hosts with a functional RDR1. Differential analysis of 21 and 22 nt read alignments revealed a subset of capsicum and *N. benthamiana* WA transcripts in which small RNA coverage increased by orders of magnitude following infection by TSWV (>10-fold increase; *p* < 10^−8^; capsicum: 1084 transcripts; *N. benthamiana* WA: 4093 transcripts; Figure 3A) or CaCV (>10-fold increase; *p* < 10^−8^; capsicum: 2307 transcripts; Figure S4A). Importantly, the same global shift in abundance of small RNAs targeting endogenous genes was not apparent in either the TSWV- or CaCV-infected *N. benthamiana* LAB accession that lacks a functional RDR1. 

To determine whether the generation of vasiRNAs was prevalent in solanaceous species, we reanalysed small RNA read data from susceptible (Marglobe) and resistant (Red Defender) tomato varieties following TSWV infection. Like capsicum and *N. benthamiana* WA, abundant vasiRNAs were also observed in TSWV-infected Marglobe tomato (Figure S4B), while these were largely absent in Red Defender. In contrast to Red Defender and its Marglobe counterpart, *N. benthamiana* LAB did not show a diminished viral load relative to WA (Figure S5B,C), indicating a decoupling of vasiRNA production from viral load in this instance. 

A subset of vasiRNA generating host transcripts was selected for further analysis based on abundant 21 and 22 nt read alignments in TSWV inoculated capsicum and *N. benthamiana* WA samples and very low alignments in their mock-inoculated counterparts (Figure 3A). Alignment profiles to *Heat Shock Cognate 70-2* (*HSC70-2*)*, Calnexin 1* (*CNX1*), and *Next to BRCA 1* (*NBR1*) in TSWV- and CaCV-infected capsicum and *N. benthamiana* WA displayed the distribution of 21 and 22 nt hotspots in both orientations (Figure 3B and Figure S4A). Minimal alignments to the same transcripts in *N. benthamiana* LAB were evident. In the reanalysed tomato samples, vasiRNA profiles with alignment hotpots were apparent for the *HSP70 heat shock protein 70* (*HSP70*) and *Calnexin* transcripts in Marglobe but not in Red Defender where vasiRNAs were absent (Figure S4B). 

### 3.5. Expression Analysis of Host Transcripts Targeted by vasiRNAs

**Figure 4:**
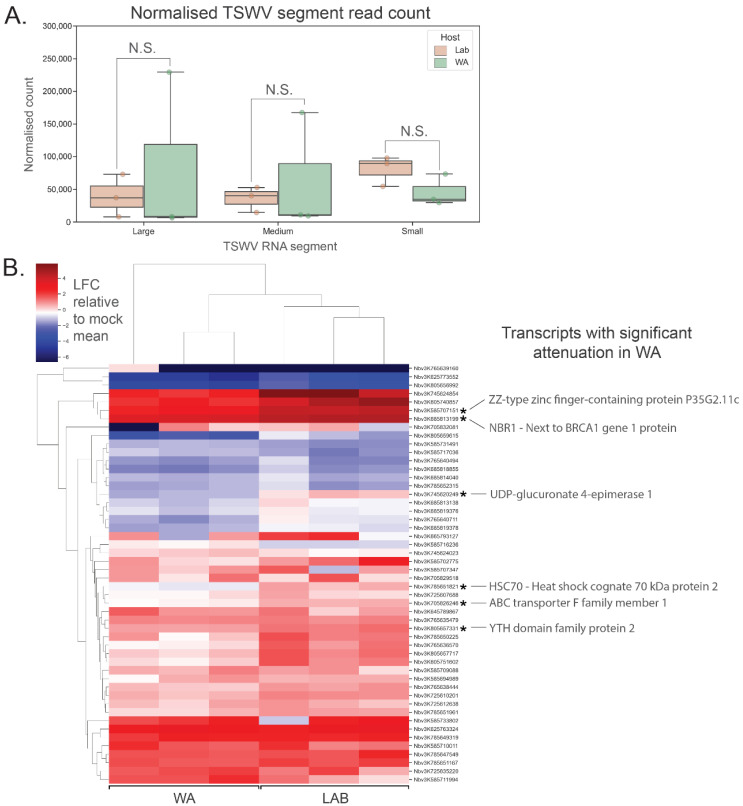
RNA-seq analysis of putative vasiRNA target transcripts in *N. benthamiana* shows inconsistent attenuation of vasiRNA-mediated expression. (**A**) No significant difference in TSWV RNA segment (L, M and S) expression was evident between *N. benthamiana* WA and LAB 10 days post inoculation (*n* = 3 for each host; error bars represent ± 1 SD; *t*-test; 2-tailed; FDR-BH multiple correction, N.S. (not significant): *p* ≥ 0.05). (**B**) Heatmap of relative vasiRNA target gene expression in *N. benthamiana* WA and LAB. Red shading indicates upregulation and blue shading downregulation in TSWV-inoculated samples relative to the mean expression in mock-inoculated samples. VasiRNA targets were selected based on the lowest adjusted *p*-values from differential vasiRNA expression analysis using DeSeq2, without functional duplication. Of the 50 transcripts shown, six were demonstrated to have significant attenuation (*n* = 3 for each host; *t*-test; 1-tailed; FDR-BH multiple correction; LFC = log transformed TSWV/mock proportional expression; alternate hypothesis = mean LFC is lower in WA compared to LAB).

Using an RNA-seq approach, viral genome segments were identified in the resulting datasets. There were no significant differences in the abundance of the three TSWV genome segments between inoculated *N. benthamiana* LAB and WA samples, though the WA samples (*n* = 3) were more variable (Figure 4A). Next, host vasiRNA-targeted transcripts were quantified, and their attenuation status was determined on a transcript-by-transcript basis. An attenuated transcript was identified as showing either greater downregulation or reduced upregulation in vasiRNA-generating WA samples compared to vasiRNA-free LAB samples following TSWV infection. A heatmap of relative host transcript abundance is shown in Figure 4B, with normalised abundances of selected targets shown in Figure S6A–E. In this analysis, the 50 transcripts with the most aligning vasiRNAs were selected, with no duplication of functional annotation. When comparing Log Fold Change (LFC) of these transcripts relative to the mock inoculation mean, no overarching trend of vasiRNA regulation of gene expression was evident. Of the transcripts analysed, the degree of upregulation was significantly reduced for six transcripts in WA compared to LAB, with 44 showing no significant differences (1-tailed *t*-test; *n* = 3 for each treatment). Expression levels of individual targets were also analysed via RT-qPCR for *N. benthamiana* WA and LAB, and additionally, capsicum (Figure S5G,H). Similar results to the RNA-seq analysis were evident for *N. benthamiana*, with abundant vasiRNAs not significantly resulting in attenuation in the WA genotype compared to vasiRNA-deficient LAB genotype. For capsicum, up- and downregulation of vasiRNA target genes occurred for both TSWV- and CaCV-inoculated treatments relative to mock across two experimental repeats. 

### 3.6. Functional Examination of Host Transcripts Targeted by vasiRNAs

**Figure 5:**
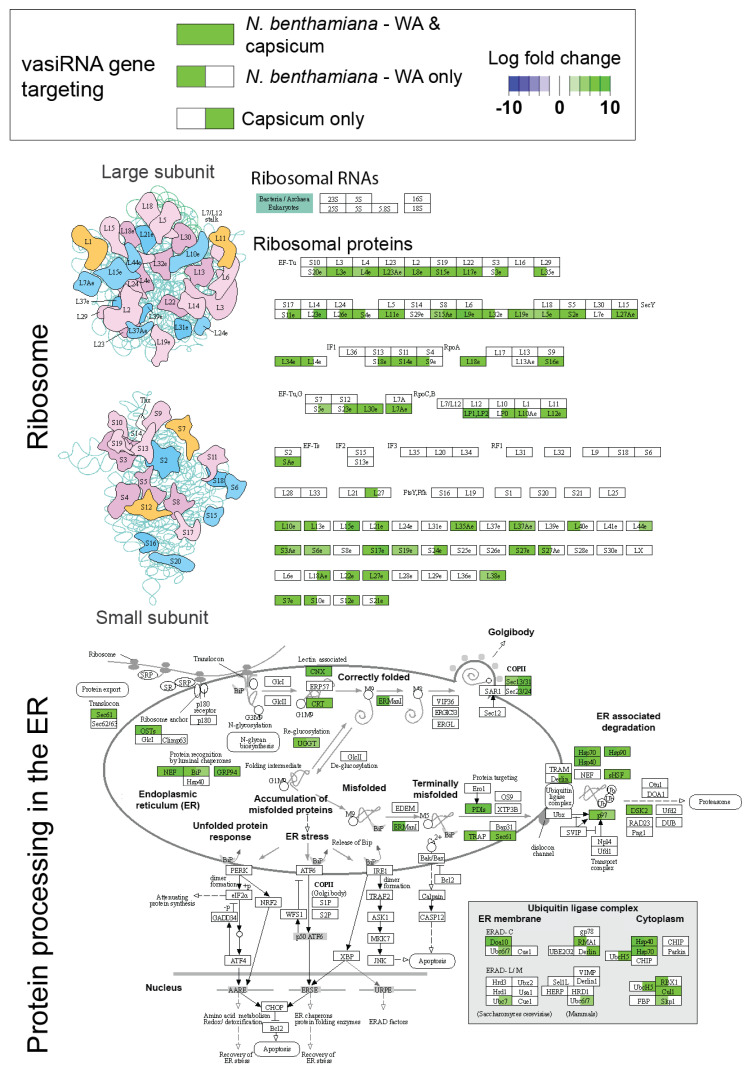
Capsicum and *N. benthamiana* WA vasiRNA targets are enriched in the ribosome and protein processing in the endoplasmic reticulum (ER) KEGG pathways. Log fold changes of significantly differentially regulated vasiRNAs (21 nt and 22 nt combined) are shown for one or both of capsicum and *N. benthamiana* WA (green shading indicates increased significantly abundance of vasiRNAs targeting associated host transcripts in response to TSWV infection, and blue shading indicates significantly decreased vasiRNA abundance). Enrichment for these pathways suggests that localisation of vasiRNA template to the ER may be the functional basis for vasiRNA target ‘selection’. KEGG pathway analysis and visualisation were carried out using Pathview, v1.36.0 (Charlotte, NC, USA) [26].

To understand why certain transcripts were targeted by vasiRNAs, a literature review of selected target genes, along with their roles in responding to pathogens, was performed (Table S1). Functional analyses were expanded to vasiRNA targets more broadly, with KEGG pathway analysis revealing vasiRNA-generating transcripts were involved in a diverse array of pathways, with genes in the cytoplasm-situated ‘ribosome’ and ‘protein processing in ER’ pathways strongly enriched (Figure 5). Both these pathways are expected to be highly active during viral replication when viral messenger RNAs are translated to proteins that are folded and modified into their final state. In order to determine whether vasiRNA biogenesis does indeed take place in the cytoplasm, small RNA reads from TSWV-infected Marglobe tomato were aligned to the well-annotated tomato genome, with no mismatches allowed. No alignments to introns were evident for the vasiRNA-generating transcripts analysed, indicating that vasiRNA generation does not take place in the nucleus, at least prior to splicing (Figure S7).

## 4. Discussion

Confirming previous reports for tospovirus-infected Solanaceae [9,10], our current work demonstrates that TSWV or CaCV infection of susceptible capsicum and *N. benthamiana* accessions leads to the generation of abundant vsiRNAs and upregulation of the associated RNAi pathway genes. The increase in 21–22 nt small RNA reads derived from infected host plants can be partly attributed to the production of vsiRNAs. Notably, a similar overall change away from 24 nt and towards 21–22 nt small RNAs in response to TSWV infection has previously been reported in peanut [8], indicating a common response across plant families. Though conserved among higher plants, this RNAi response is on its own insufficient to eliminate the virus from the host plant, although it may extend its lifespan. Notably, transgenic approaches to engineer tospovirus resistance that strongly and constitutively express viral dsRNA sequences have proven highly effective [30], indicating that the RNAi pathway induced by TSWV and CaCV does have the capacity to control virus infection effectively if the circumstances permit. Presumably, without transgenic ‘priming’, the tospovirus gains a foothold and eventually overwhelms the plant’s silencing mechanisms, possibly because of the silencing suppressor action of NSs. NSs are considered to be involved in the sequestration of vsiRNAs and preventing them from entering the RNA-induced silencing complex, thus decoupling the presence of vsiRNAs from target transcript degradation [31]. It is apparent that as viral load increases, vsiRNAs similarly proliferate as more viral transcripts become available as templates for RDR-mediated amplification. The susceptible tomato variety Marglobe has previously been shown to accumulate far more vsiRNAs aligning to the TSWV genome upon infection than the resistant Red Defender, supporting the association between vsiRNA response and viral load [9]. 

Differences in relative TSWV and CaCV vsiRNA abundance for tospovirus RNA segments and genes were evident between hosts. Abundant vsiRNAs were, however, still generated in the functional RDR1 lacking *N. benthamiana* LAB genotype, suggesting another RdRp, likely RDR6, may be sufficient for secondary vsiRNA production. The length of siRNAs derived from longer dsRNAs is determined by Dicer-like (DCL) enzyme processing, with DCL1 and DCL4 principally generating 21 nt siRNAs, DCL2 generating 22 nt siRNAs, and DCL3 generating 24 nt siRNAs in plants [29]. Though studies in *Arabidopsis* have shown that DCL4-mediated 21 nt siRNAs generated from dsRNA hairpins are significantly more abundant than their DCL2-mediated 22 nt counterparts [32,33], similar levels of 21 nt and 22 nt vsiRNAs were generated in capsicum and *N. benthamiana*. If the enhanced potency of systemic silencing induced by 22 nt siRNAs in *Arabidopsis* also occurs in the Solanaceae, it does not appear to result in reduced viral load, particularly in the *N. benthamiana* WA/TSWV pathosystem. 

Despite similar symptoms, viral loads and accumulation of vsiRNAs in *N. benthamiana* LAB and WA accessions 10 days post-inoculation, TSWV and CaCV infection subsequently proved lethal to the LAB plants, whereas the WA plants survived, albeit in a weakened state. It has previously been demonstrated that the introduction of transgenic *RDR1* into *N. benthamiana* LAB prevents lethality caused by tobacco mosaic virus (TMV) but does not prevent the systemic spread of the virus [34]. Furthermore, depletion of potato (*Solanum tuberosum*) *RDR1* transcripts does not affect susceptibility to potato virus X or TMV, as measured by the level of viral coat protein [35]. Given that *N. benthamiana* WA has a functional RDR1, this supports the notion that its presence is the determinant of enhanced survival or at least a significant contributor. 

Apart from acting together with RDR6 in the production of vsiRNAs, RDR1’s role in generating vasiRNAs has been proposed to contribute to enhanced plant longevity under viral challenge [11,15,16,18]. Capsicum and *N. benthamiana* WA generate large numbers of vasiRNAs against a specific subset of endogenous transcripts, while *N. benthamiana* LAB does not, providing further support for RDR1’s role in vasiRNA biogenesis. Profiles of vasiRNA alignments to target transcripts show the presence of hotspots in both orientations, similar to vsiRNA alignments to viral RNAs. Many endogenous vasiRNA targets identified in earlier reports for Brassicaceae and Solanaceae [11,15,16,18] have functional homologues in the vasiRNA-targeted transcripts we identified in the tospovirus-Solanaceae pathosystem, suggesting a conserved response among higher plants to multiple viruses. 

Our findings pose the questions, what impact do vasiRNAs have on transcript expression, and what is the mechanism for the selection of this subset of host transcripts? It has been suggested that vasiRNA-targeted genes are associated with successful virus infection and are thus silenced by the host to resist the virus [11]. Viruses themselves may modulate the expression of host transcripts, with the downregulation of transcripts involved in photosynthesis in response to vasiRNAs identified in Brassicaceae [16]. Our data similarly indicate that these associations are multifaceted; vasiRNA targets such as *NBR1* are associated with virus defence, though others act as important components for virus replication or systemic movement through the plant (Table S1).

Importantly, the effect of vasiRNAs on the expression of their target transcripts is nevertheless unclear. Our data do not show a clear and consistent association between vasiRNA abundance and expression of their target transcripts in TSWV-infected *N. benthamiana*. Examination of fifty representative vasiRNA-targeted transcripts without functional duplication showed that the upregulation or downregulation of these transcripts in response to TSWV infection generally occurred in both the LAB and WA genotypes. If vasiRNAs were leading to attenuation of target transcript expression in the WA genotype where vasiRNAs were present, this attenuation was generally subtle and not easily detected in the transcript expression analyses conducted. There may be several reasons for this outcome, which contrasts with the previous literature for different pathosystems [11,15,16,18]. The TSWV NSs gene, which is a suppressor of silencing, may sequester vasiRNAs, preventing them from silencing target transcripts but not impacting their biogenesis and subsequent detection in small RNA sequencing. The timing of post-inoculation tissue sampling, as well as the selection of sampling tissue type (apex/apical leaf tissue), may mean that vasiRNA-mediated gene expression impacts had occurred but were not detected. Additionally, differences other than a functional RDR1 between the two *N. benthamiana* genotypes may be confounding, serving to obscure any post-transcriptional downregulation that may be occurring. Translational inhibition is another mechanism by which protein expression could be reduced independent of transcript abundance. Indeed, 22 nt siRNAs have been shown to inhibit the translation of endogenous transcripts in response to environmental stress [36] and could be performing a similar role in virus-impacted plants. The potential disconnect between vasiRNA expression and transcript expression knockdown will be the subject of further work.

To shed light on the conserved selection of vasiRNA-target transcripts, we examined the expression of these transcripts as well as their putative functions and cellular localisation. The vast range of expression of these transcripts suggests that high expression itself in response to tospovirus infection is not how this subset of genes is selected. KEGG analysis revealed that many vasiRNA-associated transcripts were either ribosomal protein-encoding transcripts or encoded proteins located in the ER that are involved in protein processing and folding. For instance, both *CRT/CNX* and *BiP*, identified in our study as vasiRNA targets, sequester unfolded proteins at the ER and re-direct them for re-folding [37]. It is possible that co-localisation of host mRNAs and viral transcripts could lead to the production of vasiRNAs, with mRNA destined for translation at the ER possibly providing a substrate for an ER-localised RDR1 complex. Supporting this hypothesis, the alignment of small RNA reads to the tomato genome demonstrated that vasiRNAs do not align to intronic sequences, suggesting that biogenesis occurs after splicing and likely after export to the cytoplasm. Although the presence of RDR1 at the ER has not been demonstrated, reports in *Arabidopsis* show that a membrane-bound complex containing aminophospholipid transporting ATPases (ALAs) with flippase activity is essential for RDR6, RDR1, and vasiRNA production during CMV infection [12,13]. Many viruses target the ER and associated machinery for replication [38]. Indeed, localisation of TSWV to the ER during cell-to-cell movement through the plasmodesmata, along with association with membrane compartments during the virus lifecycle stages, has been demonstrated [39]. Whether co-localisation with viral transcripts acts to initiate vasiRNA priming will also be the subject of further work. 

## 5. Conclusions

When taken together, these findings demonstrate the evolutionary persistence of the host gene targeting vasiRNA biogenesis pathway, though its function in tospovirus-Solanaceae interactions remains unclear. Abundant virus-derived vsiRNAs targeting TSWV and CaCV transcripts are apparent in multiple Solanaceous species, including *N. benthamiana* LAB, which lacks vasiRNAs and a functional RDR1. The interplay between plant viruses and their hosts remains an active area of investigation, with this work showing that plant responses involving RNAi and the generation of vsiRNAs and vasiRNAs are conserved, with the functional differences apparent between different pathosystems a possible focus for enhancing crop protection. 

## Data Availability

All raw sequence data is available at the NCBI Sequence Read Archive under BioProject ID PRJNA784690.

## Supplementary Materials

The following supporting information can be downloaded at: https://www.mdpi.com/article/10.3390/pathogens11070745/s1, Figure S1: Tospovirus and mock inoculated plants used for small RNA and mRNA sequencing; Figure S2: *N. benthamiana* LAB and WA plants 23- and 47-days post TSWV and mock inoculation; Figure S3: Small RNA sequencing analysis of CaCV inoculated capsicum and *N. bethamiana* LAB; Figure S4: Abundant alignment of 21 nt and 22 nt reads to host transcripts 10 days post tospovirus inoculation suggests the presence of vasiRNAs in capsicum and *N. benthamiana* WA in response to CaCV and tomato (cv. Marglobe) in response to TSWV; Figure S5: Quantitative real-time PCR and ELISA analysis of expression of tospovirus segments, RNAi genes and vasiRNA target transcripts; Figure S6: Analysis of RNAi gene and vasiRNA target transcript expression in TSWV and mock inoculated *N. benthamiana* Lab and WA hosts (10 DPI) using RNA-seq; Figure S7: Small RNA reads do not map to tomato (Marglobe) introns of vasiRNA target genes (*HSC2-like* and *ZZ type zinc finger domain-containing protein),* suggesting the mRNAs act as templates after splicing and outside of the nucleus; Table S1: Literature search for vasiRNA targets and host-pathogen interactions; Table S2: List of qPCR primers used in this study.
