# Supplementary material for: Tospoviruses Induce Small Interfering RNAs Targeting Viral Sequences and Endogenous Transcripts in Solanaceous Plants
Source: Pathogens. 2022 Jun 30;11(7):745. doi: 10.3390/pathogens11070745 (PMC9317859; doi:10.3390/pathogens11070745)
Supplement: Supplementary file 1 [file pathogens-11-00745-s001.zip › pathogens-1770689-supplementary.pdf]

Supplementary Material

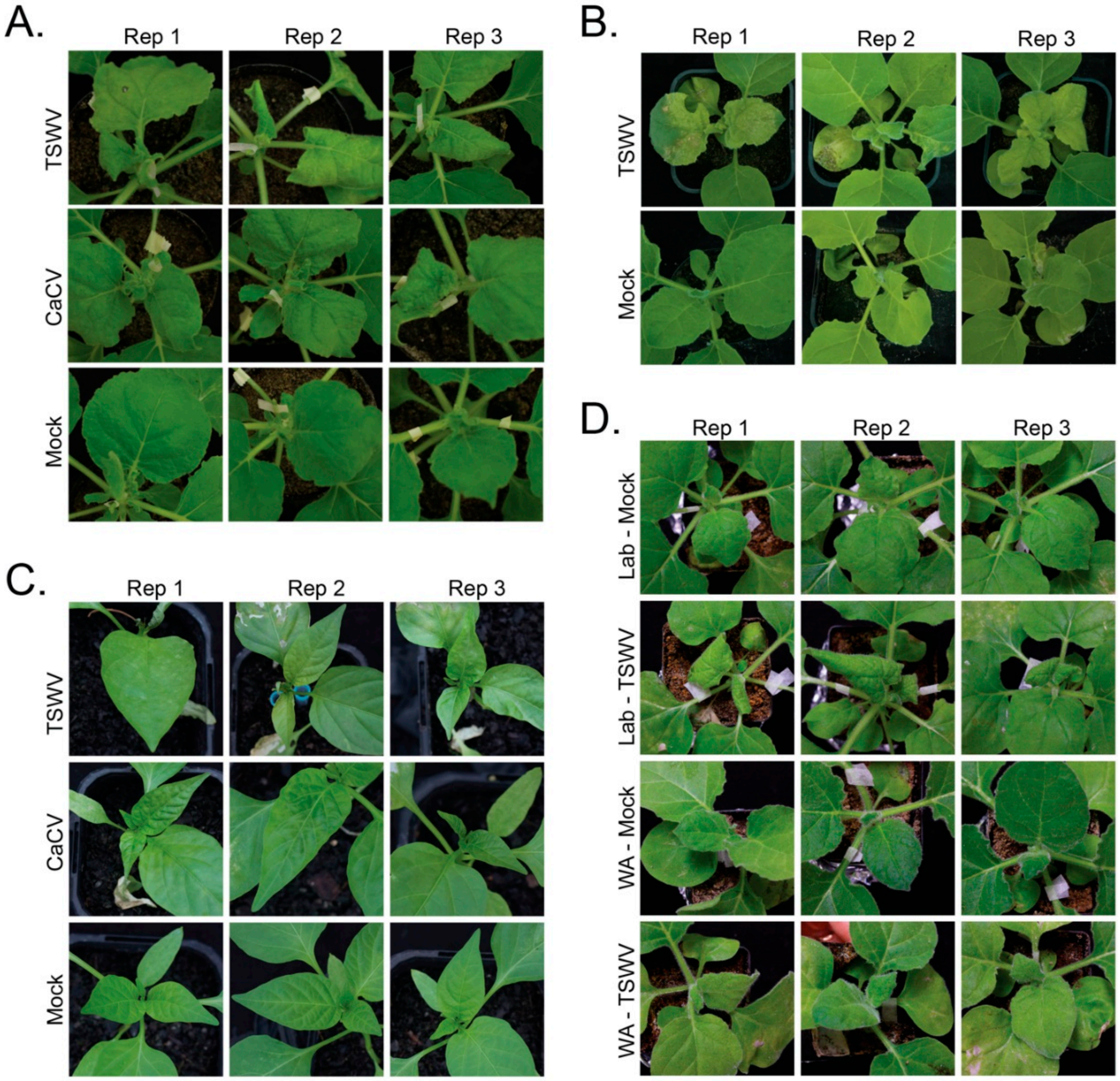

Figure S1. Tospovirus and mock inoculated plants used for small RNA and mRNA sequencing. All plants are 10-days post inoculation. All tospovirus inoculated samples showed mild symptoms at this time point. **A.** *Nicotiana benthamiana* LAB inoculated with tomato spotted wilt virus (TSWV), capsicum chlorosis virus (CaCV) or mock inoculated (n = 3 for each treatment). **B.** *N. benthamiana* WA plants inoculated with TSWV or mock inoculated (n = 3 for each treatment). **C.** Capsicum plants inoculated with TSWV, CaCV, or mock inoculated (n = 3 for each treatment). Samples derived from these plants (A, B and C) were used for small RNA sequencing and qPCR. **D.** *N. benthamiana* LAB and WA plants inoculated with TSWV or mock inoculated (n = 3 for each treatment). Samples derived from these plants were used for RNA sequencing and subsequent gene expression analysis.

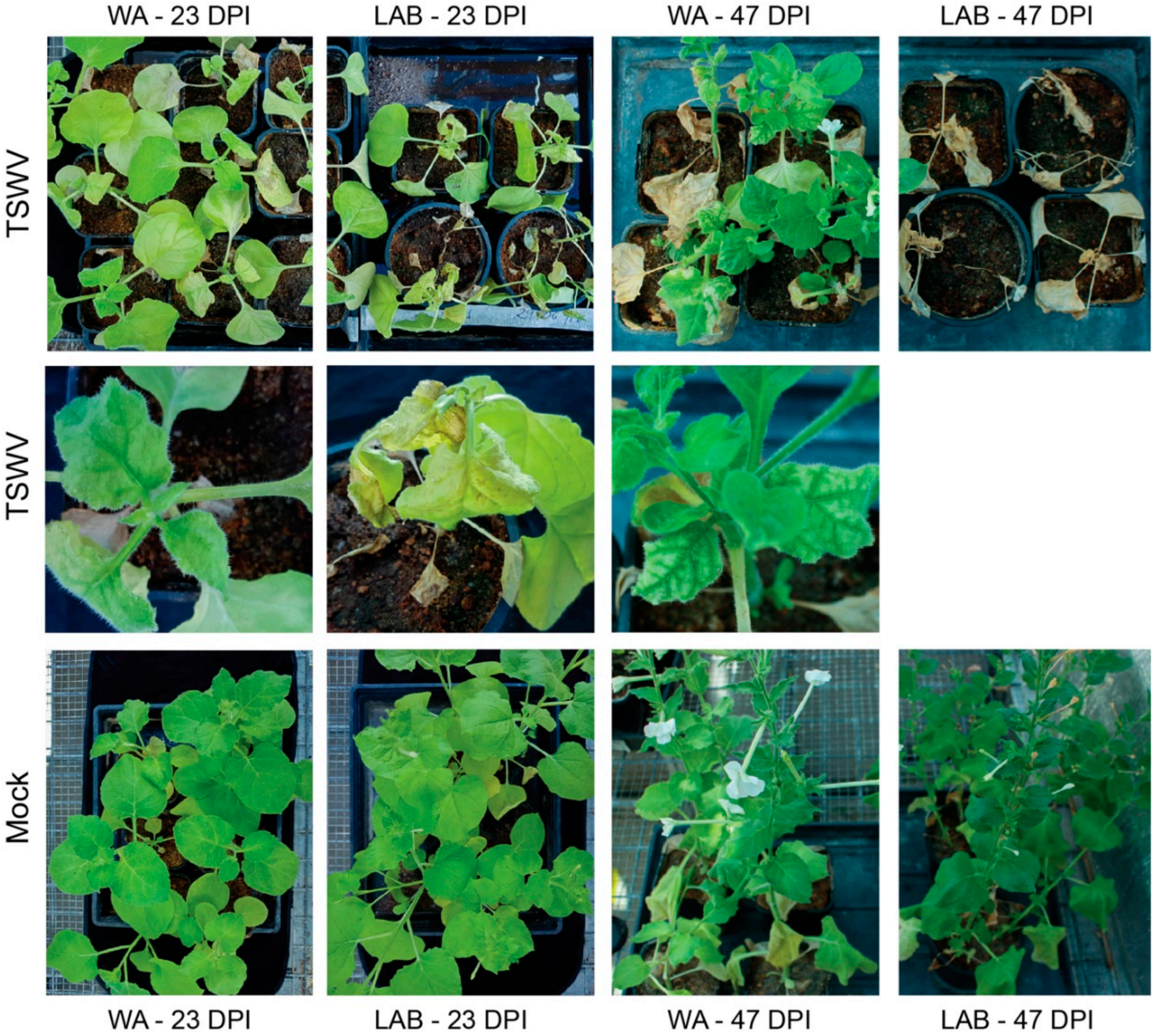

Figure S2. *N. benthamiana* LAB and WA plants 23- and 47-days post TSWV and mock inoculation. *N. benthamiana* LAB plants have more evident disease symptoms and show greater wilting at 23 DPI and are perished 47 DPI. *N. benthamiana* WA plants show strong TSVW symptoms at 47 DPI but remain alive. Mock inoculated plants are healthy at both time points.

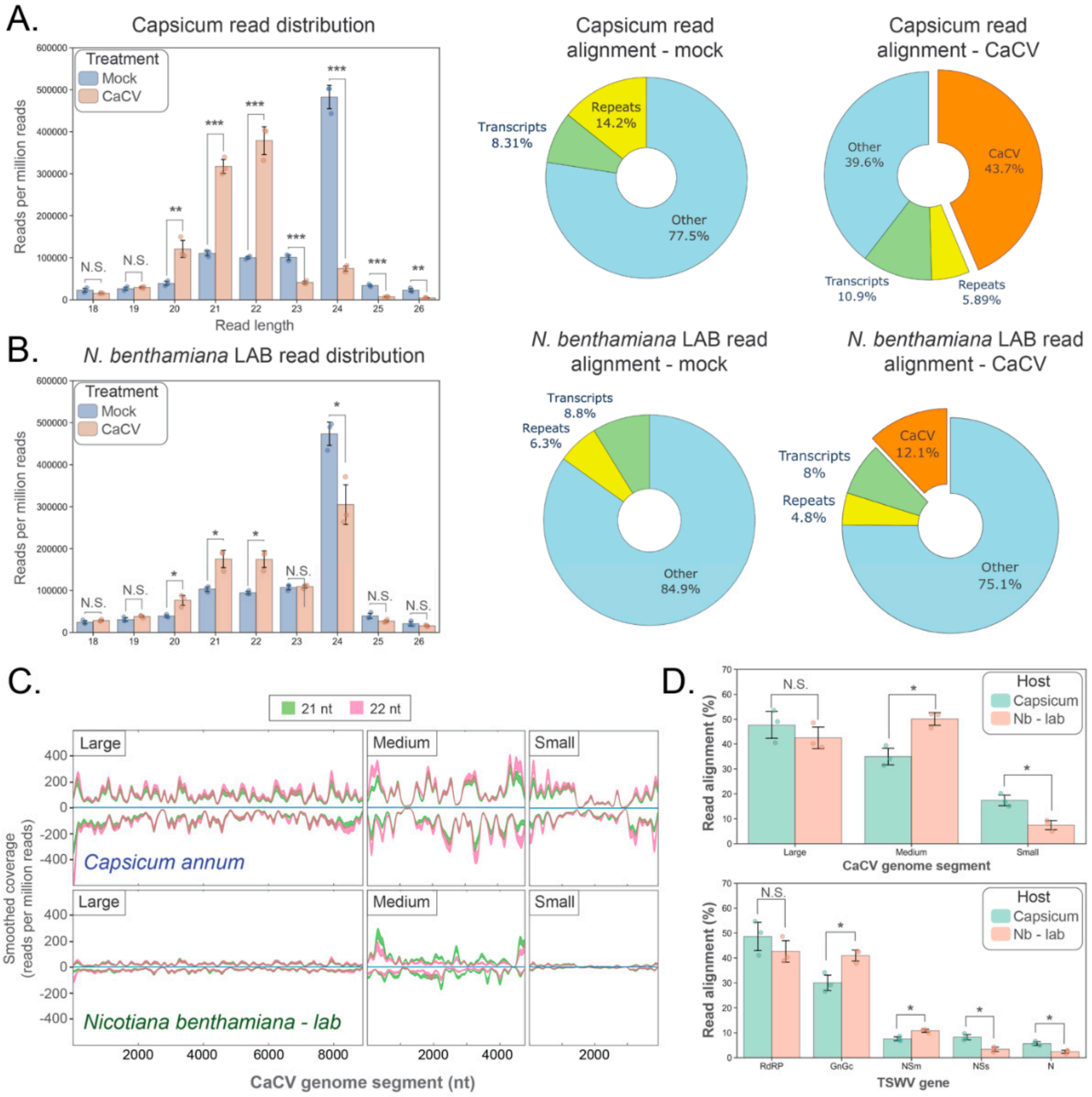

Figure S3. Small RNA sequencing analysis of CaCV inoculated capsicum and *N. bethamiana* LAB**.** As with TSWV infected hosts, a significant reduction in 24nt reads and an increase in 21 and 22 nt reads has occurred 10 DPI in **A.** capsicum and **B.** *N benthamiana* LAB (left panels; *t* test; 2-tailed; FDR-BH multiple correction). In each case, CaCV aligning reads make up a large portion of all small RNA reads (right panels; percentages are the mean of three replicates). C. Significantly more reads proportionally align to the M RNA segment of CaCV in *N. benthamiana* LAB compared to capsicum, and fewer align to the S RNA segment. Correspondingly, significantly more reads proportionally align to the *GnGc* and *NSm* genes (present on the M RNA segment) of CaCV in *N. benthamiana* LAB compared to capsicum, and fewer on the *N* and *NSs* genes (present on the S RNA segment) (Bar graphs: n=3 for each treatment; error bars represent ± 1SD; *t* test; 2 tailed; FDR-BH multiple correction; *: p<0.05, **: p<0.01, ***: p<0.001).

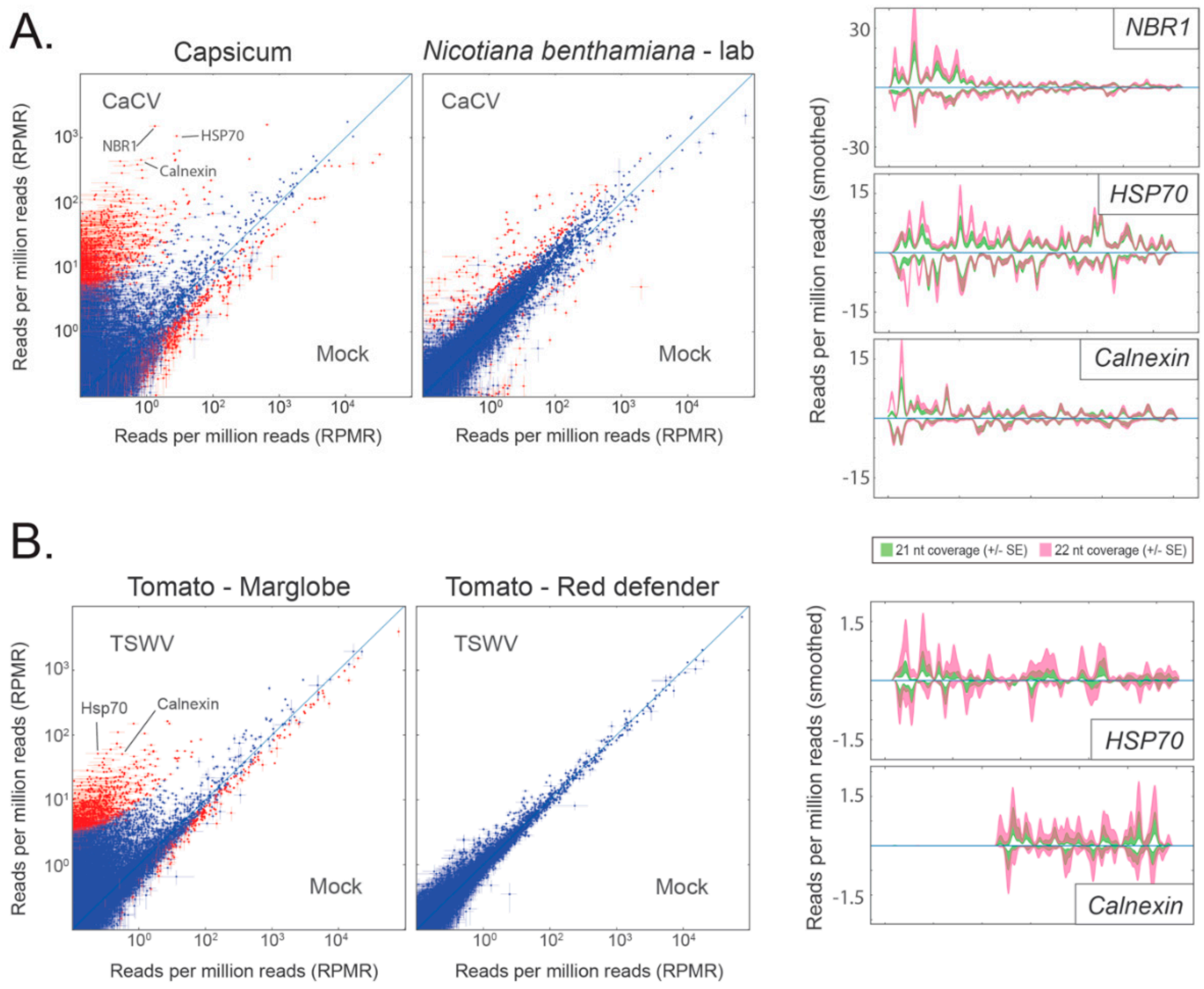

Figure S4. Abundant alignment of 21nt and 22nt reads to host transcripts 10 days post tospovirus inoculation suggests the presence of vasiRNAs in capsicum and *N. benthamiana* WA in response to CaCV and tomato (cv. Marglobe) in response to TSWV. **A.** The putative CaCV-induced vasiRNA responses in capsicum and *N. benthamiana* WA are similar those present in TSWV inoculated samples (left panel). 21nt and 22nt alignment hotspots are also present in both orientations of the *NBR1*, *HSP70* and *Calnexin* example transcripts. **B.** Re-analysis of small RNA sequencing data from Olaya et al. (2020) indicates putative vasiRNAs are generated in the TSWV suspectable tomato variety Marglobe, but not in the resistant variety Red Defender. *HSP70* and *Calnexin* are also show alignment hotspots in both orientations in Marglobe.

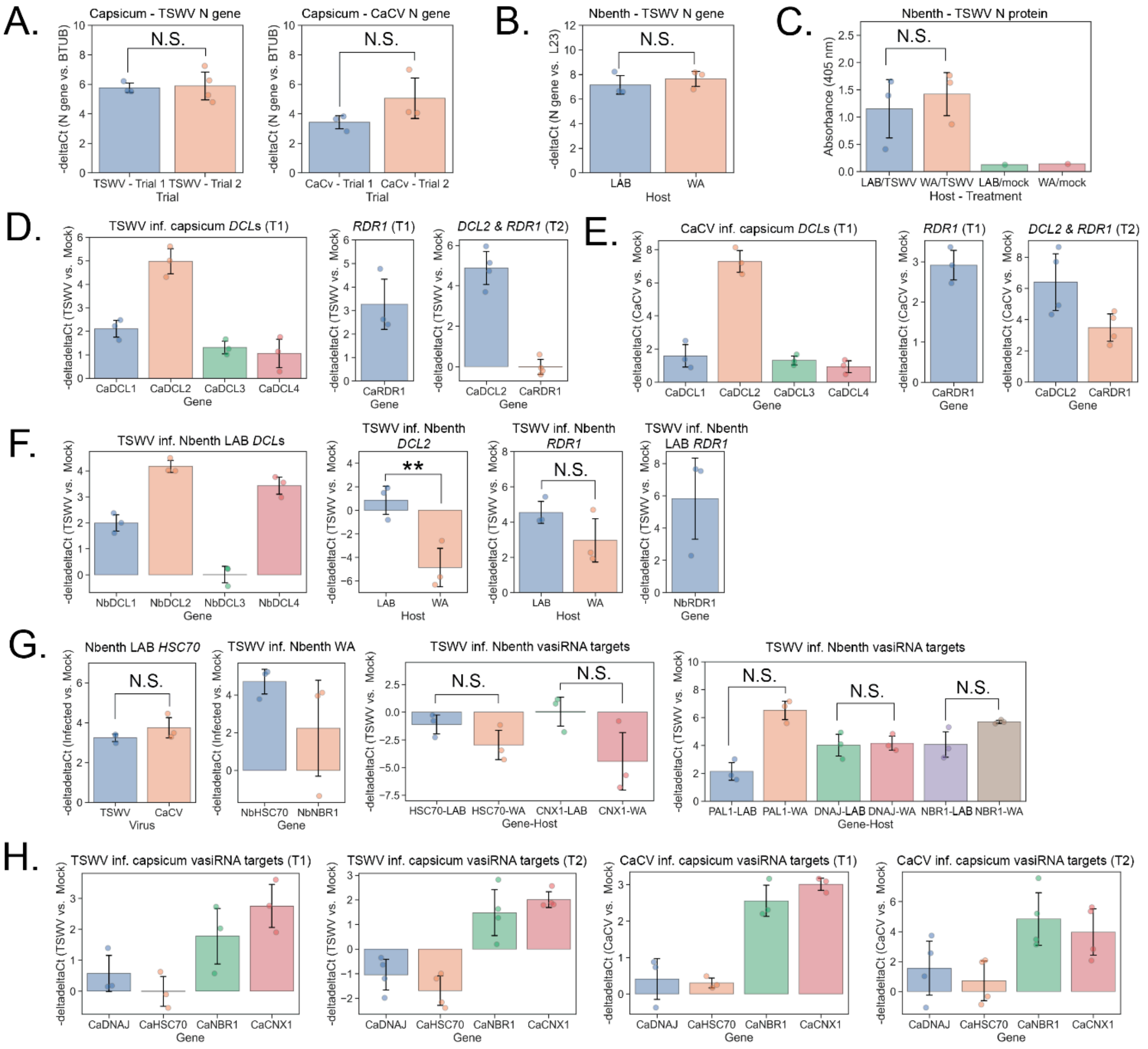

Figure S5. Quantitative real-time PCR and ELISA analysis of expression of tospovirus segments, RNAi genes and vasiRNA target transcripts. **A.** N gene abundance was not significantly different in two TSWV and CaCV capsicum inoculation trials (10 DPI; *n* = 3 or 4), indicating similar virus titre levels across trials. **B.** TSWV N gene abundance was not significantly different in *N. benthamiana* LAB and WA 10 DPI. **C.** ELISA detection of the N protein supported the qPCR outcome, with no significant difference between *N. benthamiana* LAB and WA absorbance levels. Key RNAi genes were generally upregulated in **D.** TSWV inoculated and **E.** CaCV inoculated compared to mock inoculated capsicum plants across two trials (T1 and T2; *n* = 3 or 4). Separate qPCR runs are shown in discrete boxes. **F.** RNAi genes were also generally upregulated in *N. benthamiana* LAB for TSWV inoculated plants compared to mock inoculated. *DCL2*, which generates 22nt siRNAs, was significantly downregulated in *N. benthamiana* WA compared to LAB. There was no significant difference in *RDR1* expression. **G.** Expression of vasiRNA targets. There was no significant difference in *HSC70* upregulation in *N. benthamiana* LAB following either TSWV or CaCV inoculation compared to mock (left panel). *HSC70* and *NBR1* were both upregulated in TSWV-inoculated *N. benthamiana* WA compared to mock. There was no significant difference in the regulation of *HSC70* and *Calnexin* (centre) and *PAL1*, *DNAJ* and *NBR1* (right) between *N. benthamiana* LAB and WA (TSWV inoculated compared to mock; *n* = 3 for each host). **H.** Regulation in vasiRNA targets in capsicum in response to TSWV (left panels) and CaCV (right panels) inoculation relative to mock over two trials (T1 and T2). Generally, targets were upregulated, except for *DNAJ* and *HSC70* in TSWV inoculated plants in T2. (Unless otherwise stated, *n* = 3 for each bar; individual data points shown; error bars represent ± 1SD; *t* tests were 2 tailed with FDR-BH multiple correction; *: $p<0.05$, **: $p<0.01$, ***: $p<0.001$)

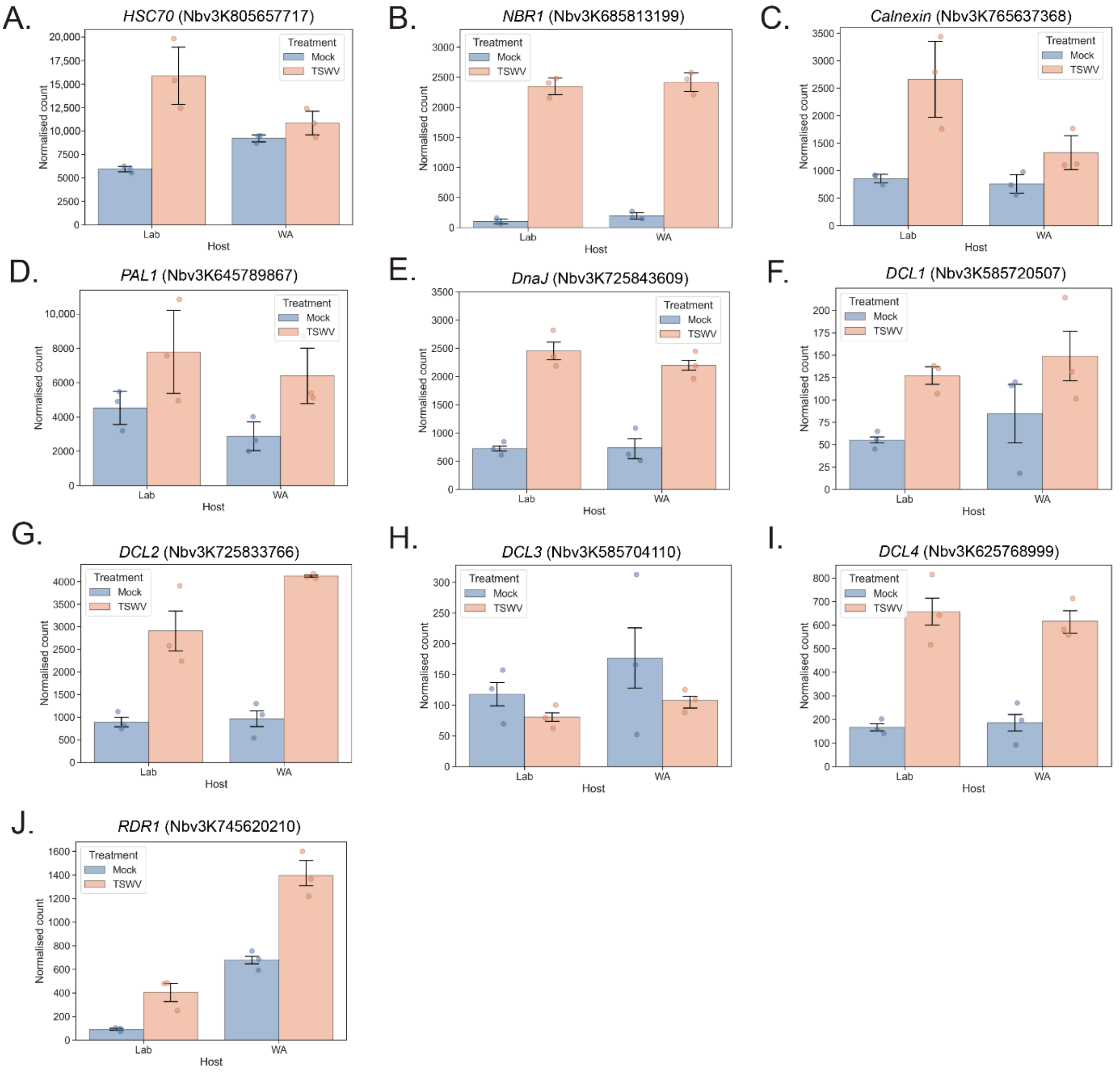

Figure S6. Analysis of RNAi gene and vasiRNA target transcript expression in TSWV and mock inoculated *N. benthamiana* Lab and WA hosts (10 DPI) using RNA-seq. Data are normalised using DeSeq2 (*n*=3 for each host; individual data points are shown; error bars represent ± 1SD). VasiRNA targets **A**. *HSC70* **B**. *NBR1* **C**. *Calnexin* **D**. *PAL1* and **E**. *DnaJ* are upregulated compared to mock in both LAB and WA. RNAi genes **F**. *DCL1* **G**. *DCL2* **H**. *DCL3* **I**. *DCL4* and **J**. *RDR1* are also upregulated in both LAB and WA.

HSC2-like

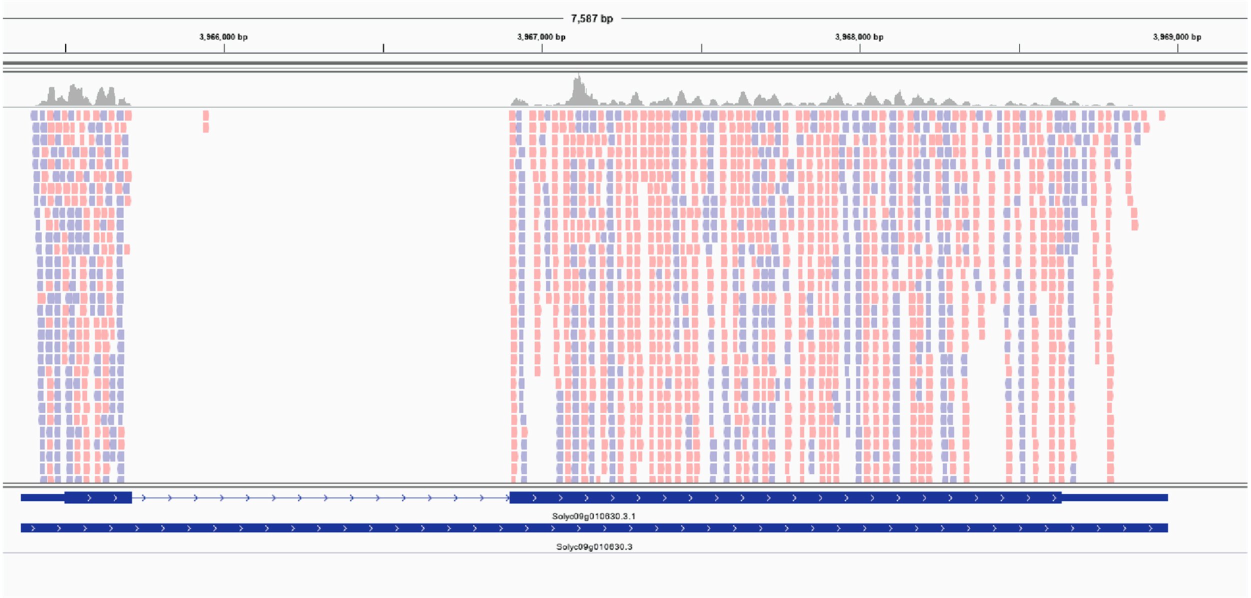

ZZ type zinc finger domain-containing protein

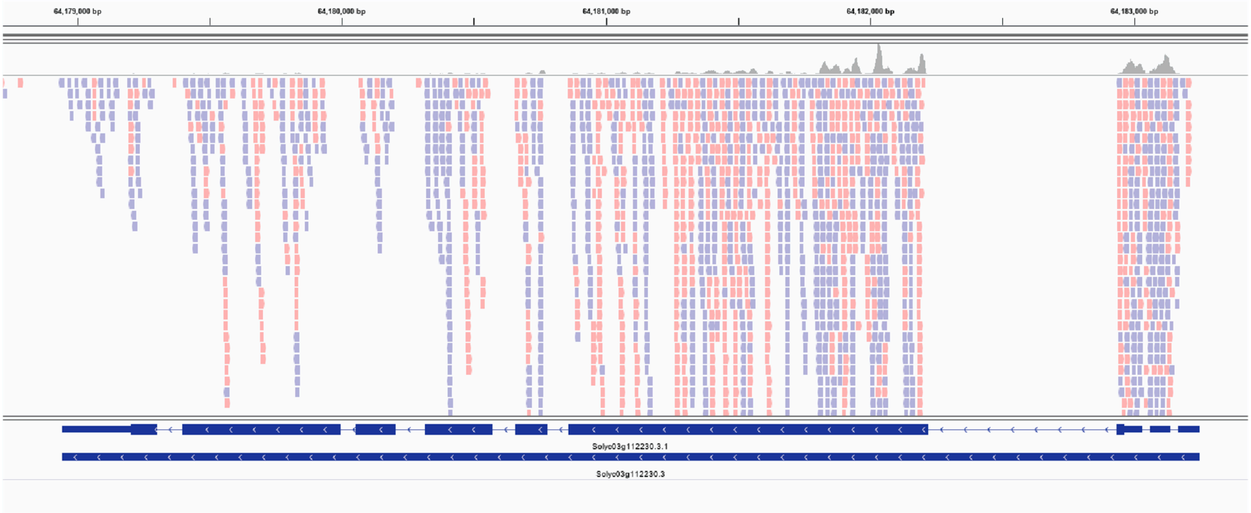

Figure S7. Small RNA reads do not map to tomato (Marglobe) introns of vasiRNA target genes (*HSC2-like* and *ZZ type zinc finger domain-containing protein)*, suggesting the mRNAs act as templates after splicing and outside of the nucleus. Tomato was used for this analysis due to its well structurally annotated genome. Aligned read pairs are stacked. Exons are shown by thick blue lines with chevrons at the bottom of each plot. Introns are shown as fine blue lines with chevrons. There are little to no small RNA reads aligning to introns in either target.

Table S1. Literature search for vasiRNA targets and host-pathogen interactions**.**

Gene	Experimental host	Pathogen or stress	Mechanism	Reference
Heat Shock Cognate 2 (HSC70-2)	*A. thaliana*	TuMV or TCV	Upregulation in systemically infected tissue. Response to protein accumulation in the cytosol.	Aparicio *et al*., 2005
	A. thaliana	TuMV	HSP interaction with virus RNA-dependent RNA polymerase (RdRP). Potential to form component of virus replication complex.	Dufresne *et al*., 2008
	N. benthamiana	TuMV, TCV, TVCV and TRV	Upregulation in infected tissue. Response to protein accumulation in cytosol.	Aparicio *et al*., 2005
	N. benthamiana,	CNV	Associates with particle and required for particle disassembly.	Alam and Rochon, 2017
	A. thaliana	*Pseudomonas syringae*	Upregulation in response to Pst infection. *HSC70-1* interacts with SGT1 and regulates plants immune and environmental responses. SGT normally mediates protein degradation of target proteins and binds to resistance genes such as NB-LRR.	Noel *et al*., 2007
	Solanum lycopersicum	PepMV	HSC70 upregulated and interaction with PepMV coat protein	Mathioudaki *et al*., 2012
	S. lycopersicum	TYLCV	Virus induces formation of protein aggregates containing HSP70 and reducing the quantity in the cytosol. Reduced HSP70 in the cytosol triggers upregulation of *HSP* transcripts.	Gorovits and Czosnek
	Frankliniella occidentalis	TSWV	Upregulation of HSP70 in TSWV infected thrips vector	Ogada *et al*., 2017
DnaJ	*N. tabacum* and *A. thaliana*	TSWV	DnaJ family member accumulates in response to TSWV infection and homologs interacts with movement protein (NSm).	Bargen *et al*.2001, Soellick *et al*. 2000
	N. benthamiana	PVX	DnaJ interacts with PVX nucleocapsid protein and stem-loop of RNA genome. DnaJ inhibits replication and movement of PVX	Cho *et al*., 2012
Neighbor of BRCA1 (NBR1)	*A. thaliana*	CaMV	Targets CaMV capsid protein for autophagic degradation and required for host resistance.	Hafren *et al*., 2017
Phenylalanine ammonia lyase 1 (PAL1)	*N. tabaccum*	TMV	Upregulation in response to TMV infection. Key enzyme in the metabolism of secondary metabolites such as salicyclic acid and others involved in defence, such as capsaicin.	Pellegrini *et al*., 1994, Kim and Hwang, 2015

Table S2. List of qPCR primers used in this study.

Gene target	Species	Primer name	Sequence 5' to 3'	Reference
Beta tubulin (βTUB)	*C. annuum*	CaBetaTUB-For	GAGGGTGAGTGAGCAGTTC	Wan et al., 2011
		CaBetaTUB-Rev	CTTCATCGTCATCTGCTGTC	Wan et al., 2011
CaCV N gene	*Capsicum Chlorosis Virus (CaCV)*	CaCV N-F1	AGCAACTTGACAGCCTGTTTGA	This study
		CaCV N-R1	GGCCTTGCACCAATTATTTTCCC	This study
Calnexin 1 (CNX1)	*C. annuum*	CaCNX1_FWD	GAAGCCGGCTAGAGCTAGTG	This study
		CaCNX1_REV	GCGAGTGCCTTACCAATCCT	This study
	N. benthamiana	NbCNX1_FWD	AGGATGGGCTTGAATGTGGT	This study
		Nb_CNX1_REV	GGCTCCGCATTTGTCTGGT	This study
Dicer-like 1 (DCL1)	*C. annuum*	CaDCL1-F	AAAGGAAAGAGTTGGTCTATCGG	This study
		CaDCL1-R	CTCTTTCTGGCATGTTCAA	This study
	N. benthamiana	qNbDCL1-F	AAAAGAATGAGATGGTATTTCGG	Katsarou et al., 2016; Dadami et al., 2013
		qNbDCL1-R	TTCTTTCTGGCATGCTCAA	Katsarou et al., 2016; Dadami et al., 2013
Dicer-like 2 (DCL2)	*C. annuum*	CaDCL2-F	TGAAGGAGAATCACTTCTTAGGG	This study
		CaDCL2-R	CGAGGCCATAACCAAGGACTC	This study
	N. benthamiana	qNbDCL2-F	GAAGAACCACTTCTTAGGGGAAA	Katsarou et al., 2016; Dadami et al., 2013
		qNbDCL2-R	GGCCATAACAAGGACTCAA	Katsarou et al., 2016; Dadami et al., 2013
Dicer-like 3 (DCL3)	*C. annuum*	CaDCL3-F	AGAACTGCATCATTTGTGGG	This study
		qNbDCL3-R	GGCCACAACAACCATGTCCT	Katsarou et al., 2016
	N. benthamiana	qNbDCL3-F	AGGACTGCAGCGTTTATGGT	Katsarou et al., 2016
		qNbDCL3-R	GGCCACAACAACCATGTCCT	Katsarou et al., 2016
Dicer-like 4 (DCL4)	*C. annuum*	CaDCL4_F	GCACTCACTACAGAGAACTGCCATG	This study
		CaDCL4_R	ACAATGTTCGAGCGCTTTCT	This study
	N. benthamiana	DCL4_Fmod	GCACTTACTACAGAGAAATGCAATG	This study. Modified primer from Katsarou et al., 2016; Kotakis et al., 2010
		DCL4_Rev_b	ACAATGTTTGAGCGCCTTCT	Katsarou et al., 2016; Kotakis et al., 2010
DnaJ homolog 2 (DnaJ2)	*C. annuum*	CaDnaJ_FWD	TAGACGAGTGCGAGGAGACT	This study
		CaDnaJ_REV	TCATATGCCTCTTGGGCAGC	This study
	N. benthamiana	NbDnaJ_FWD	ATTCTACAATTTGCTGGGTGCG	This study
		NbDnaJ_REV	ATCGCCAAGCTTCAACATGC	This study
F-box	*N. benthamiana*	F-BOX-F	GGCACTCACAAACGTCTATTTC	Katsarou et al., 2016; Liu et al., 2012
		F-BOX-R	ACCTGGGAGGCATCCTGCTTAT	Katsarou et al., 2016; Liu et al., 2012
Glyceraldehyde-3-phosphate dehydrogenase (GAPDH)	*C. annuum*	CaGAPDH-For	ATGATGATGTGAAAGCAGCG	Wan et al., 2011
		CaGAPDH-Rev	TTTCAACTGGTGGCTGCTAC	Wan et al., 2011
Heat shock cognate 2 (HSC70-2)	*C. annuum*	CaHSC70-2-5'endFWD	AACCTCTTTGCTGCCTCTCC	This study
		CaHSC70-2-5'endREV	GTTGCCATACACCAACGCAG	This study
	N. benthamiana	NbHSC70-2-5'endFWD	AACCCATTTGCCGCCCCTCC	This study
		NbHSC70-2-5'endREV	CAAACTCCAACGCAAGAGTAC	This study
L23	*N. benthamiana*	L23-F	AAGGATGCCGTGAAGAAGATGT	Katsarou et al., 2016; Liu et al., 2012
		L23-R	GCATCGTAGTCAGGAGTCAACC	Katsarou et al., 2016; Liu et al., 2012
Neighbour of BRCA1 (NBR1)	*N. benthamiana*	NbNBR1_FWD	ATGGTTGTGGTGTTCATCCG	This study
		NbNBR1_REV	AACCTCGCAGCATGCAGATC	This study
	C. annuum	CaNBR1_FWD	ATGGTTGTGGGGTTCATCCG	This study
		CaNBR1_REV	CGAAACCTTCCATGCAGAGC	This study
Phenylalanine lyase 1 (PAL1)	*N. benthamiana*	NbPAL_FWD	TGATCTCGTCCCCTTGTCCT	This study
		NbPAL_REV	GGGTTTCGCCATTAGGTCCA	This study
RNA-dependent RNA Polymerase 1 (RDR1)	*C. annuum*	CaRDR1_FWD	ATGCAGAGGCCATTGGTGTTGCTG	This study
		CaRDR1_REV	CCAAGCTGAAGCCTTTGGTAACAT	This study
	N. benthamiana	CaRDR1_FWD	ATGCAGAGGCCATTGGTGTTGCTG	This study
		NbRDR1_REV	CCAAGCCGAAGCCTTCGCTAACAT	This study
TSWV N gene	*Tomato Spotted Wilt Virus (TSWV)*	TSWV N-F1	TTTGGGTCAATCCCGAGGTC	This study
		TSWV N-R1	AAAGTCTGTGAGGCTTGCCA	This study
